# Comparison of Drying Shrinkage of Concrete Specimens Recycled Heavyweight Waste Glass and Steel Slag as Aggregate

**DOI:** 10.3390/ma13225084

**Published:** 2020-11-11

**Authors:** So Yeong Choi, Il Sun Kim, Eun Ik Yang

**Affiliations:** 1 Research Institute for Disaster Prevention, Gangneung-Wonju National University, Gangneung, Gangwon 25457, Korea; csy7510@gwnu.ac.kr; 2 Department of Civil Engineering, Gangneung-Wonju National University, Gangneung, Gangwon 25457, Korea; iskim@gwnu.ac.kr

**Keywords:** fundamental properties of steel slag, heavyweight waste glass, drying shrinkage, prediction model

## Abstract

This study analyzed the fundamental properties of concrete using steel slag, to test its viability as an aggregate material. An experimental investigation into the effect of steel slag as a coarse aggregate, and heavyweight waste glass as a fine aggregate, on the drying shrinkage of concrete was performed. The calculated shrinkage strain was compared to five different shrinkage prediction models, namely, the ACI 209, B3, KCI 2012, EC 2 and GL 2000 model codes, to evaluate their ability to accurately predict shrinkage behavior. From the results, the elastic modulus of concrete increased with the increase in steel slag substitution ratio, however drying shrinkage decreased. The predictive value of the existing prediction model of drying shrinkage differed from the experimental values, and requires correction to improve its accuracy. The B3 model code showed the best prediction results of drying shrinkage.

## 1. Introduction

Concrete is composed of cement, mixing water, and aggregates which constitute approximately 80 percent of the total mass [1]. Increased demand for concrete has led to the depletion of natural aggregates [2,3]. Therefore, there is an increasing need to find alternative materials to replace natural aggregates. One possible solution is to replace the natural aggregates with various types of industrial waste [4,5]. Generally, industrial waste is either discarded or buried in a landfill, which leads to environmental hazards. Several types of industrial waste are now used in the manufacture of eco-friendly materials, replacing the traditional construction materials [6]. When the analog TV broadcasting system was converted to a digital TV broadcasting system in Korea, a large volume of funnel glass containing a number of heavy metals, such as lead, iron, and others, was discarded as waste [7]. Various studies showed that this heavyweight waste glass can be used as an ingredient in concrete without fundamentally changing its characteristics [8,9,10,11,12,13]. It has been extensively studied for many years, and its use as fine aggregate in concrete has resulted in several benefits, such as an improved resistance to drying shrinkage and increased durability [8,13,14,15].

Korea is the fifth largest producer of steel in the world, and generates a large amount of steel slag. Steel slag is an industrial waste and is obtained as a by-product during the production of steel [16]. Extensive research is underway on recycling steel slag into a high-value material, which can lead to significant economic benefits [17]. Additionally, its use as aggregate in concrete has resulted in advantages such as improved resistance to shrinkage [17,18]. However, it is not suitable for reuse as an aggregate in concrete, since it contains free CaO, which causes expansion and does not secure volume stability [19]. To use steel slag as an aggregate, the free CaO content must be reduced to below 2% to prevent expansion, otherwise it cannot be used [20]. Previously, free CaO content in steel slag could not be reduced to under 2%, and it was mainly used as an aggregate for sub-base coarse materials in Korea. The specifications for steel slag were constituted to evaluate its applicability for soil compaction [21]. However, when steel slag is aged, the probability of expansion or collapse can reduce [22]. To age the steel slag, it is exposed to air for at least three months, which transforms the CaO into Ca(OH)_2_, stabilizing it. Aging the steel slag in water can significantly reduce the aging period [23].

Drying shrinkage is defined as a volume change due to the drying of concrete; it affects the stability of concrete, and is dependent on the properties of concrete-making materials and mix proportions [24,25]. Therefore, the characteristics of drying shrinkage must be evaluated when different materials are used in concrete.

As described above, the mechanical properties and the durability of concrete (mortar) using heavyweight waste glass as fine aggregate were investigated. The results show that heavyweight waste glass can be used to reduce the drying shrinkage [26]. This is due to the low water absorption value of the glass [26,27,28]. The reduction effect of drying shrinkage was more pronounced with the increase in substitution ratio of the heavyweight waste glass [9,26,29]. However, when heavyweight waste glass is substituted as a fine aggregate in concrete (mortar), the compressive strength decreases. This is most likely due to the poorer adhesion between the smooth surface of glass and the cement paste [8]. The performance degradation caused by heavyweight waste glass in concrete can be improved by using mineral admixture as a binder. Our previous studies demonstrated the feasibility of using heavyweight waste glass as a fine aggregate in concrete [8,12,13,26,27]. Until now, however, studies on the drying shrinkage of concrete using both heavyweight waste glass and steel slag have rarely been conducted. In other words, investigations into the characteristics of drying shrinkage for concrete specimens using steel slag as the coarse aggregate and heavyweight waste glass as the fine aggregate are unprecedented.

Consequently, the objective of this paper is to experimentally investigate drying shrinkage in a concrete specimen using heavyweight waste glass and a substitution ratio of steel slag. Furthermore, we compared the experimental values of drying shrinkage to the predicted values from existing models.

## 2. Materials and Methods

### 2.1. Materials

#### 2.1.1. Binder

In this study, ordinary Portland cement (ASTM Type I, i.e., OPC) was used in all the concrete specimens. To investigate the effect of mineral admixtures on drying shrinkage, parts of the cement was replaced. These were fly ash (i.e., FA) and blast furnace slag (i.e., BFS). The physical and chemical compositions of the binders are shown in Table 1.

#### 2.1.2. Coarse Aggregate

Crushed gravel was used as a coarse aggregate with a maximum aggregate size, Gmax, of 20 mm. The specific gravity and absorption ratio of the coarse aggregate were 2.68% and 0.97%, respectively. Furthermore, the material properties of coarse aggregate were measured according to ASTM C 127 [30] and ASTM C 136 [31], respectively. The material’s properties are shown in Table 2.

#### 2.1.3. Heavyweight Waste Glass

Heavyweight waste glass has a specific gravity of 3.0 and fineness modulus of 3.4. It was crushed by a jaw crusher for use as fine aggregate in concrete. Furthermore, only the crushed heavyweight waste glass that could pass through a 5 mm sieve was used, and the material properties of heavyweight waste glass were measured according to ASTM C 128 [32] and ASTM C 136 [31], respectively. The material’s properties are shown in Table 2. The physical and chemical compositions of the heavyweight waste glass are shown in Table 3; they consists of heavy metals, such as iron, lead, and chromium, etc.

#### 2.1.4. Steel Slag

**Figure 1:**
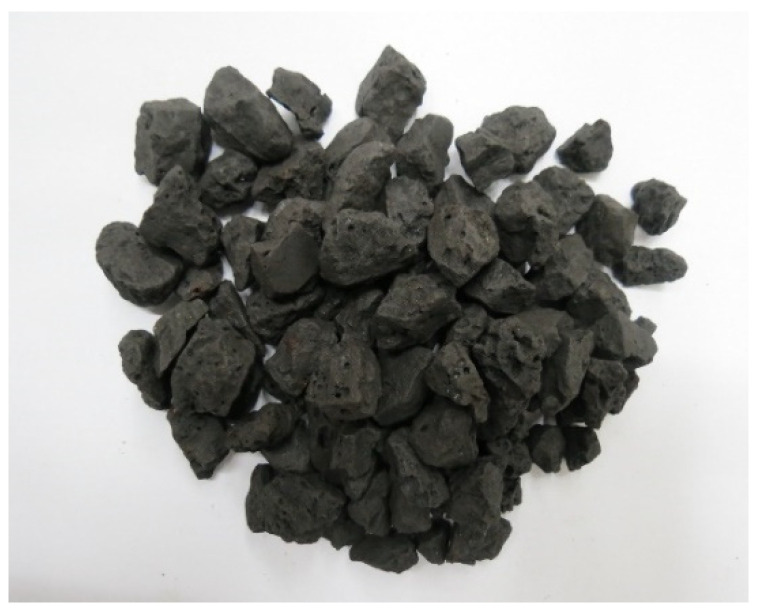
Appearance of steel slag.

**Figure 2:**
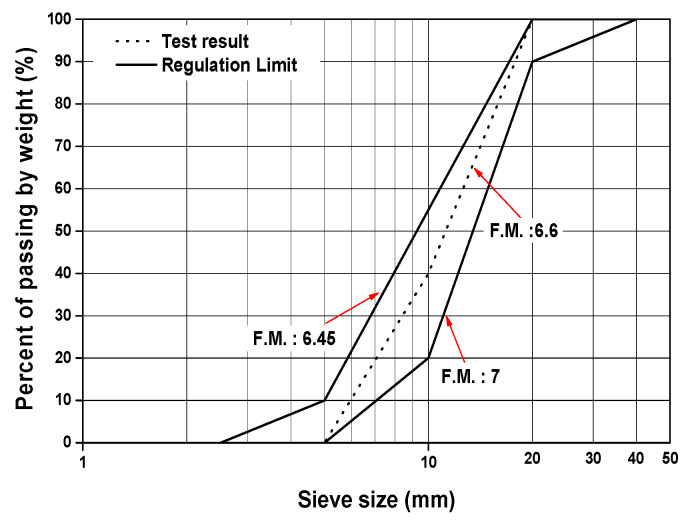
Grading curves of the steel slag.

Steel slag obtained in Korea was tested to investigate its suitability for use as coarse aggregate in concrete. The steel slag particles exhibit an angular shape, sharp edges and a porous surface texture, as presented in Figure 1. A sieve analysis was conducted on the steel slag. A standard sieve set ranging from 5 mm to 20 mm was used for the aggregate. Figure 2 shows that the grading of the steel slag was within the regulation limit. The steel slag has a specific gravity of 3.65 and fineness modulus of 6.6. The steel slag was aged in water for over a month to secure its stability.

Meanwhile, before using steel slag as coarse aggregate in concrete, its mechanical properties should be considered. This study is executed various mechanical property tests on steel slag, and the results are presented in Table 4, with both the mechanical properties of steel slag, according to KS F 2527 [33], and the specification values shown together.

In particular, to use steel slag as the aggregate in concrete, an expansion test of steel slag must be performed. The immersion expansion ratio of steel slag must be below 2% to satisfy the required specifications [20]. To measure its immersion expansion by a direct method, the steel slag is prepared, based on the JIS A 5015 [20]. According to the specifications, the steel slag is immersed in water at 80 °C, for six hours, after which the chamber temperature is gradually restored to 20 °C over eighteen hours. This process is repeated for 10 days, and then the steel slag volume is determined by using the calibrated indication (resolution: 0.01). The immersion expansion is calculated as the mean value after repeating the operation three times.

From the test results, the immersion expansion, density, and unit weight volume satisfied the specification value. However, the absorption ratio of steel slag was higher than the limit regulated by KS F 2527 [33], due to the many surface voids of the steel slag. Nevertheless, the difference in value was not excessive. The chemical composition of the steel slag is shown in Table 5, as determined using X-ray fluorescence. While CaO and MgO were below the specification value, the amount of FeO detected was slightly higher.

### 2.2. Test Variables and Mix Proportions

To investigate the drying shrinkage of concrete using steel slag as the coarse aggregate with different substitution ratios, the length change test was carried out for the concrete specimen. Heavyweight waste glass was used as the fine aggregate. The test variables and methods are listed in Table 6. 

In general, concrete needs a water-to-binder ratio of 35–55% to facilitate the mixing and to maintain its workability. Above all, based upon considerable experiments in a laboratory setting, in this study, the water-to-binder ratio of the specimen is fixed at W/B 45%. For the evaluation of the properties of the concrete, steel slag was used as a substitute for coarse aggregate at 0%, 50%, and 100% by volume. The mix proportions for the concrete specimens used in this study are shown in Table 7.

### 2.3. Test Method

#### 2.3.1. Experimental Setup for the Properties of Fresh Concrete

To investigate the fresh properties of the concrete, the slump and air contents were measured. The slump and air content experiments were executed in accordance with ASTM C 143 [34] and ASTM C 231 [35], respectively.

#### 2.3.2. Experimental Setup for the Compressive Strength and Elastic Modulus

For the compressive strength test, the specimens were prepared based on ASTM C 39 [36]. The compressive load was supplied by a universal testing machine with a capacity of 1000 kN. Each compressive strength value was an average of three measurements for each specimens. Additionally, for the determination of the elastic modulus, the linear variable displacement transducer (i.e., LVDT) was attached on the opposite side of the cylindrical specimen at a mid-height level (measuring distance: 100 mm), and the displacement value was obtained from the LVDT. The elastic modulus was calculated from the stress–strain relationship obtained. The elastic modulus of ASTM C 469 [37] was calculated.

#### 2.3.3. Experimental Setup for the Drying Shrinkage Test

The drying shrinkage of the concrete specimen (dimensions: 100 mm × 100 mm × 400 mm) was measured according to the procedures presented in ASTM C 157 [38]. Each drying shrinkage value was an average of three measurements for each specimens. The specimen for the test was cured for 7 days in limewater, and then transferred to a chamber at a temperature of 20 ± 3 °C with a relative humidity of 60 ± 5%. The strain due to drying shrinkage was automatically measured for 250 days by using embedded strain gauges, and the test results were then compared with predicted values.

## 3. Results

### 3.1. Fundamental Properties

#### 3.1.1. Slump and Air Content of Concrete with Steel Slag and Heavyweight Waste Glass

**Figure 3:**
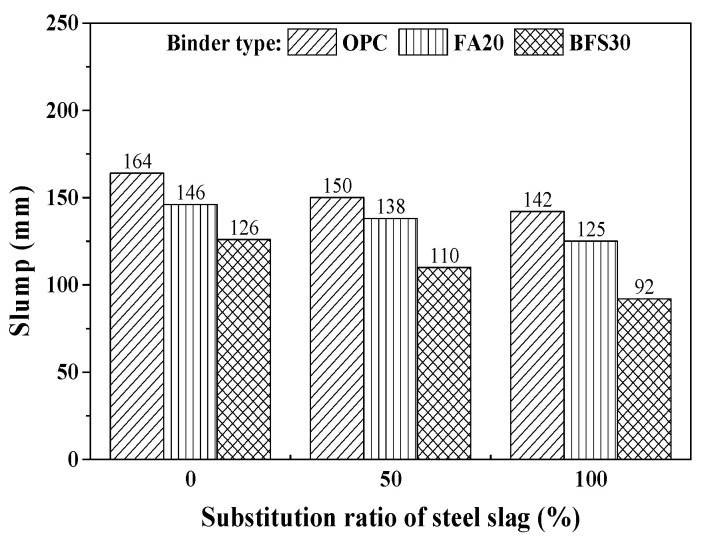
Results of slump test.

**Figure 4:**
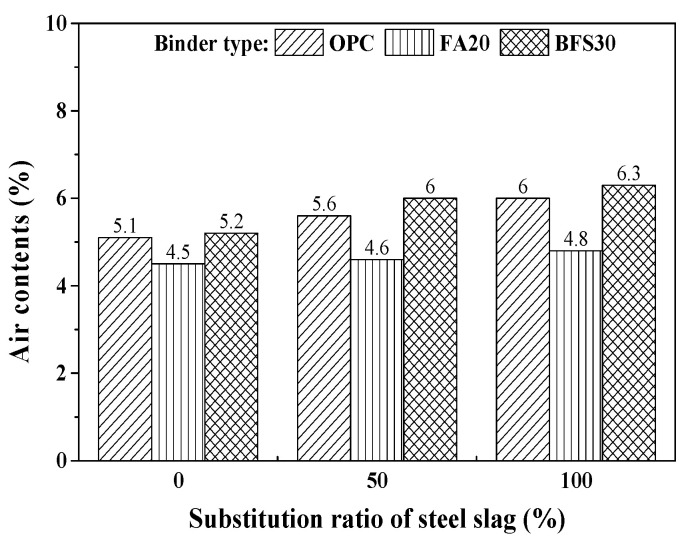
Results of air contents test.

The test results of slump and air content are shown in Figure 3 and Figure 4, respectively. Figure 3 shows the effect of the steel slag substitution ratio on the concrete slump value. The slump decreases slightly as the steel slag substitution ratio increases, regardless of the mineral admixture type. The high density of the steel slag used as coarse aggregate decreases the slump of concrete.

Figure 4 shows the effect of the steel slag substitution ratio on air content. As the substitution ratio of steel slag increases, air content increases. The steel slag with pores in the surface affects the air content of concrete.

#### 3.1.2. Compressive Strength and Elastic Modulus of Concrete with Steel Slag and Heavyweight Waste Glass

**Figure 5:**
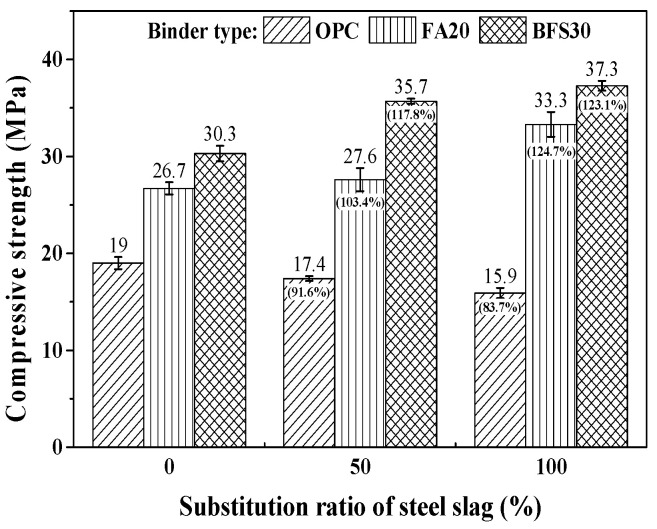
Results of compressive strength test.

The results for the compressive strength of concrete, which is a typical factor for the prediction of drying shrinkage, are shown in Figure 5, along with the relative percentage difference compared to 0%. In the case of OPC, the compressive strength decreases with the increase in the steel slag substitution ratio. This indicates the reduction in compressive strength, due to the use of heavyweight waste glass as the fine aggregate in concrete, cannot be improved by replacing steel slag as the coarse aggregate. However, in the cases with FA20 and BFS30, when the steel slag substitution ratio increases, the compressive strength also increases. This happens despite the use of steel slag as the coarse aggregate and heavyweight waste glass as the fine aggregate. This can be attributed to a pozzolanic reaction and a filler effect due to the high fineness of the mineral admixture [8].

**Figure 6:**
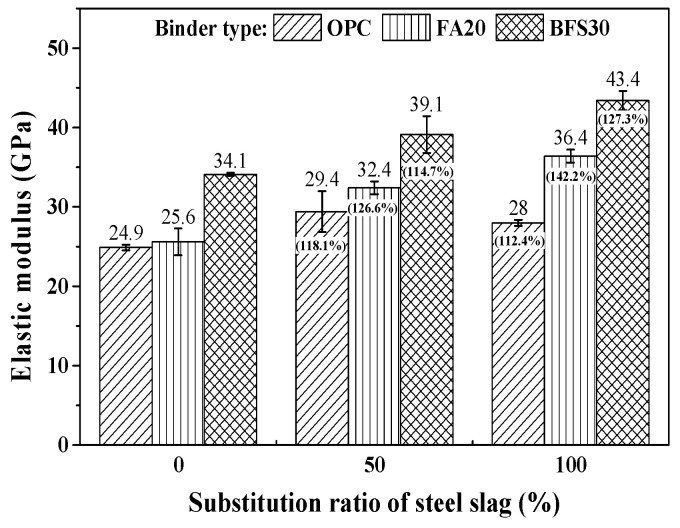
Results of modulus elastic.

The results of the elastic modulus test on the concrete substituted with steel slag and heavyweight waste glass are presented in Figure 6, along with the relative percentage difference compared to 0%. The elastic modulus increases with the increase in the substitution ratio of steel slag, by approximately 14.7–42.3%, as compared to a substitution ratio of 0%. It would appear that the higher density of the steel slag, which is an important factor affecting the elastic modulus, contributes to the increment in elastic modulus. Furthermore, the increase in the compressive strength of the concrete using mineral admixture, compared to that using OPC, also contributes to the improved elastic modulus.

### 3.2. Drying Shrinkage

#### 3.2.1. Test Results of Drying Shrinkage

**Figure 7:**
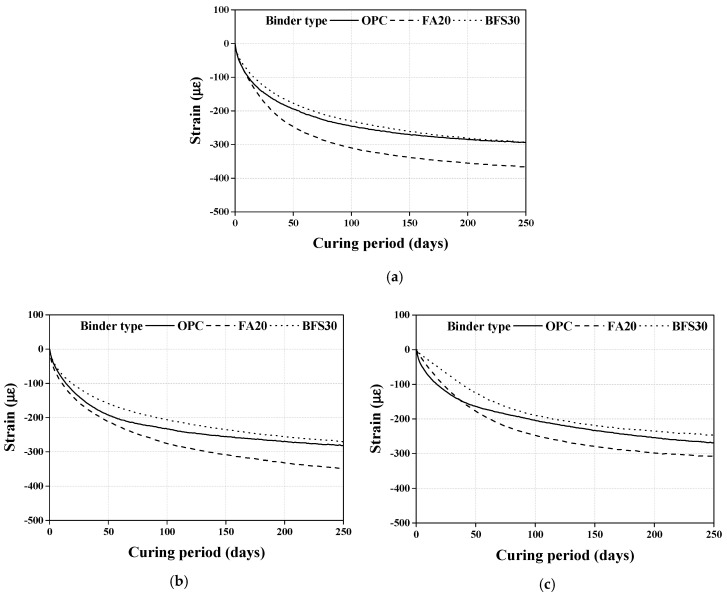
Results of drying shrinkage test. (**a**) Substitution ratio of steel slag (0%); (**b**) Substitution ratio of steel slag (50%); (**c**) Substitution ratio of steel slag (100%).

The test results of the drying shrinkage of concrete with steel slag as the coarse aggregate and heavyweight waste glass as the fine aggregate are shown in Figure 7.

The drying shrinkage of all specimens increases dramatically initially, and continues to increase up to the first 50 days of the curing period. In the later stages of the curing period, drying shrinkage slows down considerably and proceeds at a gradual rate.

The process of drying shrinkage is caused by the evaporation of water by hydrostatic tension from the small capillary pores of the cement paste and the adsorbed water from C-S-H [39]. The cement containing the mineral admixture has capillary pores smaller than 50 nm, which increases the capillary tension responsible for drying shrinkage [39,40]. Consequently, as shown in Figure 7a, the drying shrinkage is higher in cement with the mineral admixture due to the pozzolanic reaction and pore size refinement mechanism [39,40].

The drying shrinkage of the concrete specimen with steel slag as the coarse aggregate is reduced regardless of the type of mineral admixture, by approximately 4.2–16%, as compared to a substitution ratio of 0%, because the increased density due to steel slag is an important factor that influences drying shrinkage reduction [1]. Moreover, the density of the aggregate is related to its modulus of elasticity. Therefore, for concrete specimens with steel slag as the coarse aggregate, the inner stress against shrinkage increases due to the higher modulus of elasticity compared to the natural aggregate [1].

In contrast, since the drying shrinkage of concrete depends on the absorption of the aggregate, the high absorption of steel slag increases the drying shrinkage. However, the overall effect of substituting steel slag is a decrease in drying shrinkage, since it is more affected by the higher density than the higher water absorption of steel slag. Furthermore, as previously noted, using heavyweight waste glass as the fine aggregate in concrete reduces drying shrinkage because of the low water absorption of the glass. Therefore, using heavyweight waste glass and steel slag as fine and coarse aggregate, respectively, can reduce the drying shrinkage in concrete specimens.

#### 3.2.2. Comparison with Prediction Models of Drying Shrinkage

Drying shrinkage gradually occurs over a relatively long time, making it difficult to measure. There are several drying shrinkage prediction models based on experimental results, each requiring many parameters. In this study, five prediction models were selected to compare the experimental results with the prediction results of concrete specimens using steel slag as coarse aggregate and heavyweight waste glass as fine aggregate. The parameters required for the prediction of drying shrinkage in concrete by these models are tabulated in Table 8.

As shown in Table 8, all the drying shrinkage prediction models reflect the binder class, curing condition, age and humidity. However, the other factors required for drying shrinkage prediction differ slightly for each prediction model. For example, the ACI 209 model code considers the properties of fresh concrete, such as slump and air contents, because the ACI model code was built using shrinkage data from conventional concrete [41]. Additionally, the EC 2, KCI 2012 and GL 2000 models require the fewest parameters, making them easy to use.

As previously noted, drying shrinkage is strongly influenced by the properties of the coarse aggregate in concrete [1]. However, the existing prediction models do not take these properties into consideration. Consequently, there are expected differences between the predicted values and the experimental values of drying shrinkage. To understand the characteristics of the various prediction models of drying shrinkage in concrete, a comparison of the experimental results with the model prediction results is vital.

#### 3.2.3. Investigation of Effects of Model Parameters in Drying Shrinkage

**Figure 8:**
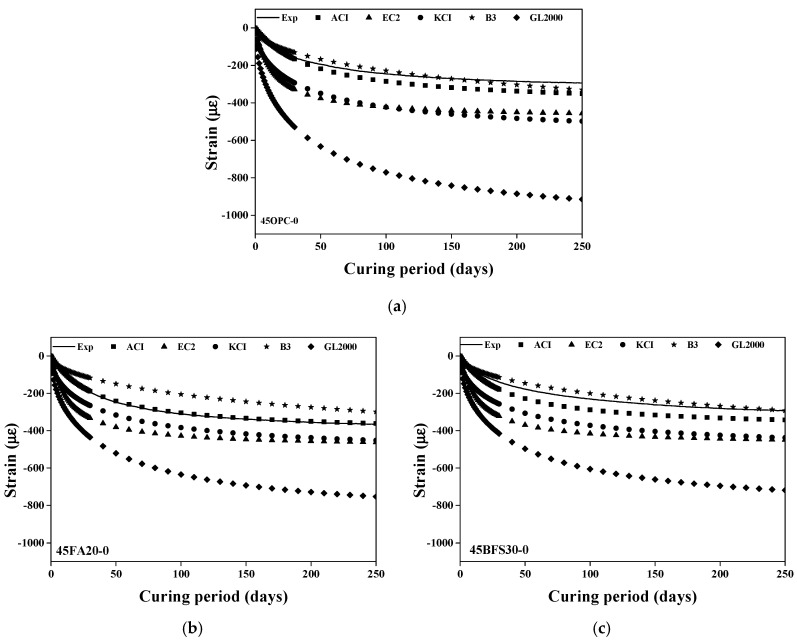
Comparison between test results and predicted value (Steel slag substitution ratio: 0%). (**a**) 45OPC; (**b**) 45FA20; (**c**) 45BFS30.

The selected prediction models were ACI-209R [42], Euro code 2 [43], KCI 2012, B3 [44], and GL 2000 [45]. Figure 8 shows the comparison between the experimental results and the prediction results of the selected models for a concrete specimen without steel slag. It is quite evident that the predicted values of drying shrinkage are not consistent with the experimental results. Since heavyweight waste glass has a low water content (0%) compared to natural aggregate, the amount of moisture in the concrete is reduced, which in turn reduces drying shrinkage. However, the existing prediction models do not reflect this, because they only consider the water-filled fine aggregate pores (saturated surface–dry aggregate). Nevertheless, the B3 and ACI models’ prediction values were the closest to the experimental results. This is because the B3 model reflects various material properties, such as moisture content, compressive strength and binder content, and the ACI model considers parameters affecting the characteristics of concrete, such as air content and slump.

The accuracy of the prediction models of drying shrinkage were in the order of B3 > ACI > KCI 2012 > GL 2000, regardless of the mineral admixture type in the initial curing stage (first 30 days). However, in the case of the EC 2 model, the predicted value of drying shrinkage after 100 days does not increase significantly when using OPC as the binder, as it uses compressive strength over long term age (91 days). As the increase in compressive strength with the OPC binder at 91 days was not greater than that with the mineral admixtures, the predicted vales of drying shrinkage converge to a certain level. Furthermore, since the EC 2 and KCI models are based on the CEB-FIP model, their predicted values of drying shrinkage are similar.

The GL 2000 model shows the highest variation from the experimental results, since it is based on actual structure and is distinctly influenced by V/S (volume surface ratio), compared to the other models. Consequently, the predicted values of drying shrinkage increase with the decrease in the size of the concrete specimen.

**Figure 9:**
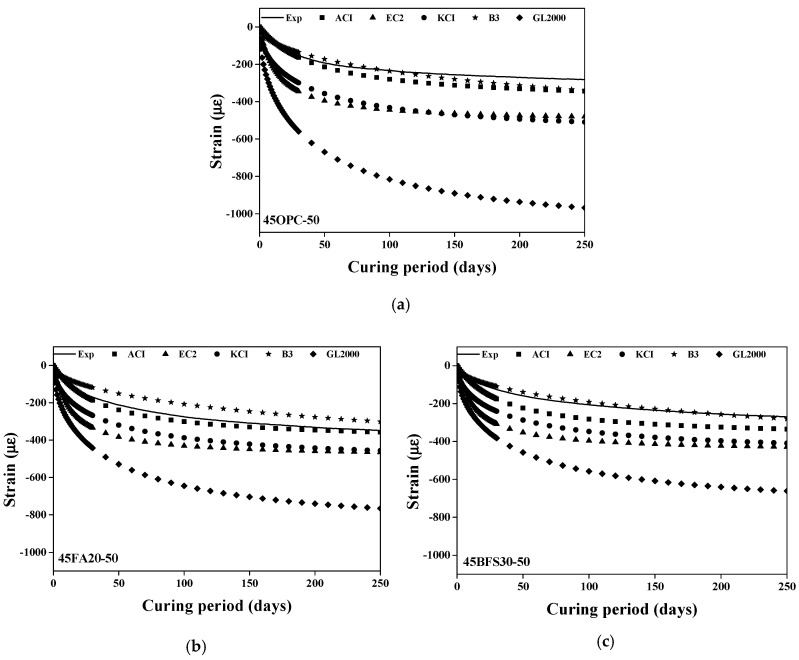
Comparison between test results and predicted value (Steel slag substitution ratio: 50%). (**a**) 45OPC; (**b**) 45FA20; (**c**) 45BFS30.

**Figure 10:**
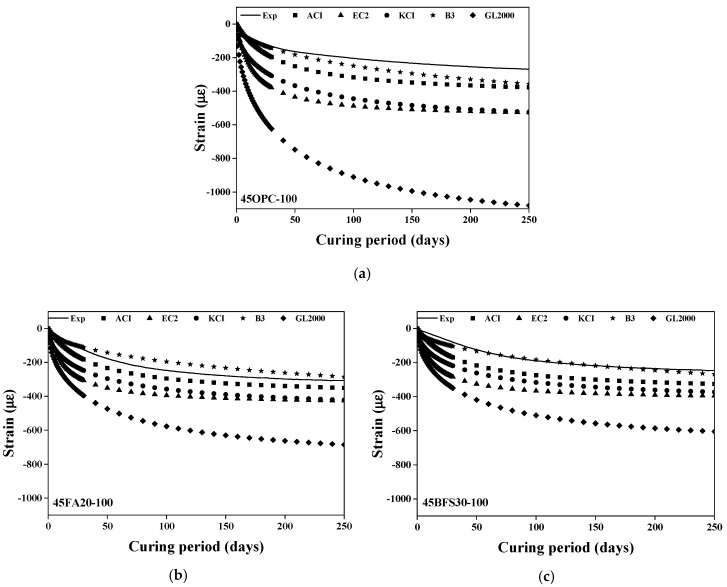
Comparison between test results and predicted value (steel slag substitution ratio: 100%). (**a**) 45OPC; (**b**) 45FA20; (**c**) 45BFS30.

Figure 9 shows the comparison of the experimental and predicted values of the drying shrinkage value of the concrete specimen using 50% steel slag as the coarse aggregate. Figure 9 shows a similar trend to Figure 8. In addition, the existing prediction models do not reflect the increase in the elastic modulus of concrete or the change in the material properties of the aggregate, such as density and elastic modulus. Therefore, the predicted values of drying shrinkage are significantly higher than the experimental results. Figure 10 shows the comparison of experimental and predicted values for the drying shrinkage of concrete with heavyweight waste glass as the fine aggregate and 100% steel slag as the coarse aggregate. The existing prediction models do not reflect the reduction in compressive strength and the increase in elastic modulus due to the steel slag aggregate when using OPC as a binder.

Unlike the other prediction models, the GL 2000 model considers the elastic modulus of the concrete specimen. However, the influence of compressive strength and V/S further increases even if the elastic modulus is taken into consideration. Consequently, the decrease in drying shrinkage due to the increase in the elastic modulus of the concrete specimen is reflected. Therefore, to effectively predict the drying shrinkage of concrete, the existing prediction models require corrections.

## 4. Conclusions

The test measurements and comparisons of the prediction models for the drying shrinkage of concrete using industrial waste as aggregate were examined. The conclusions obtained from this study are given below:

(1) When steel slag is used as the coarse aggregate in concrete, drying shrinkage decreases with the increase in the substitution ratio of steel slag, since it is affected more by the higher density than the higher water absorption of steel slag. Thus, steel slag can be used to reduce drying shrinkage in concrete.
(2) The existing prediction models do not effectively predict the drying shrinkage that is affected by the properties of the aggregate, and need to be corrected for concrete using steel slag as the coarse aggregate and heavyweight waste glass as the fine aggregate.
(3) The B3 model best predicts the drying shrinkage because it reflects various material properties such as moisture content, compressive strength and binder content. In contrast, the GL 2000 model predicted distinctly different results compared to the other prediction models.
(4) When steel slag is replaced as the coarse aggregate of concrete, the elasticity modulus can be improved, due to the high density of steel slag, by approximately 14.7–42.3%, as compared to a substitution ratio of 0%.
(5) Conclusively, to effectively predict the drying shrinkage of concrete specimens using industrial waste aggregates, a correction of the existing prediction model is required.

## Figures and Tables

**Table 1:** Physical properties and chemical compositions of the binders.

	Binder	OPC	FA	BFS
Properties				
Physical	Specific gravity	3.15	2.19	2.92
	Blaine (cm^2^/g)	3200	3200	6300
Chemical (%)	SiO_2_	21.36	51.74	33.54
	Al_2_O_3_	5.03	21.47	15.22
	Fe_2_O_3_	3.31	3.16	0.51
	CaO	63.18	1.10	43.88
	MgO	2.89	-	2.62
	SO_3_	2.30	-	2.54
	LOI	1.40	2.56	0.01

**Table 2:** Material properties of the aggregates.

Type	Density (g/cm^3^)	Absorption (%)	Fineness Modulus
Coarse	2.68	0.97	7.01
Heavyweight waste glass	3.00	0.00	3.34

**Table 3:** Physical and chemical compositions of heavyweight waste glass.

	Product	Type 1	Type 2	Type 3	Type 4	Avg.
Properties						
Physical	Specific gravity	3.0				
	Fineness Modulus	3.34				
Chemical (%)	Fe_2_O_3_	49.9	40.3	40.3	42.0	43.1
	PbO	15.1	12.7	12.7	12.8	13.3
	Cr_2_O_3_	16.7	14.4	14.4	14.4	15.0
	SiO_2_	9.6	20.4	20.4	18.7	17.3
	K_2_O	1.8	2.8	2.7	2.6	2.5
	Other	6.8	9.4	9.5	9.5	8.8

**Table 4:** Physical properties of the steel slag.

Type	Standard	Specification Value	Test Results	Standard Deviation
Property of immersion expansion	JIS A 5015 [20]	Below 2%	0.07%	±0.01%
Density	KS F 2527 [33]	3.1 g/cm^3^ and above	3.65	±0.03 g/cm^3^
Unit weight volume	KS F 2527 [33]	1.6 g/cm^3^ and above	2.2	±0.01 g/cm^3^
Absorption ratio	KS F 2527 [33]	Below 2%	2.05%	±0.01%

**Table 5:** Chemical composition of steel slag.

Type	CaO	MgO	FeO
Specification (KS F 2527) value (%)	Below 40	Below 10	Below 50
Test results (%)	24.4	0.5	51.5

**Table 6:** Test variables.

Items	Contents
W/B ratio	45%
Mineral admixture (replacement ratio)	FA (20%), BFS (30%)
Heavyweight waste glass substitution ratio	100 (%)
Steel slag substitution ratio	0, 50, 100 (%)
Fresh concrete	Slump, Air contents
Specimen size	Ø100 mm × 200 mm (Compressive strength test, Elastic modulus test)
	100 mm × 100 mm × 400 mm (drying shrinkage)
Curing condition	Water curing (20 ± 3 °C)

**Table 7:** Concrete mix proportions.

Specimen ID	W/B (%)	S.R *	Unit Weight (kg/m^3^)							Additives (Binder × %)	
			Water	Cement	H.G **	G ***	S.S ****	FA	BFS	AE Agent	S.P.
45OPC-0	45	0	170	378	851	1008	-	-	-	0.02	0.5
45OPC-50	45	50	170	378	851	504	686	-	-	0.02	0.5
45OPC-100	45	100	170	378	851	-	1373	-	-	0.02	0.5
45FA20-0	45	0	170	302	838	992	-	76	-	0.02	0.5
45FA20-50	45	50	170	302	838	496	676	76	-	0.02	0.5
45FA20-100	45	100	170	302	838	-	1351	76	-	0.02	0.5
45BFS30-0	45	0	170	265	848	1004	-	-	113	0.02	0.5
45BFS30-50	45	50	170	265	848	502	684	-	113	0.02	0.5
45BFS30-100	45	100	170	265	848	-	1367	-	113	0.02	0.5

* Steel slag substitution ratio; ** Heavyweight waste glass; *** Natural coarse aggregate; **** Steel slag.

**Table 8:** Factors of various prediction models for drying shrinkage in concrete.

Factors		ACI 209	EC 2	KCI 2012	B3		GL2000
Concrete composition	Binder	Grade, blain, and type					
		Content	-		Content		-
	Aggregate	Content	-		S/C		-
	Water	-			Content		-
Properties of concrete		Slump	Compressive strength				
		Air contents	-			Elastic modulus	
Geometry	Size and shape	Volume–surface ratio	Notional size of cross section		Volume–surface ratio		
Time	Initial Curing	Condition (moist or steam)					
		Temperature					
	After loading	Exposed to drying (age when drying begins)					
		Age of concrete					
Moisture		Relative humidity					
